# Robust Step Counting for Inertial Navigation with Mobile Phones

**DOI:** 10.3390/s18093157

**Published:** 2018-09-19

**Authors:** Germán Rodríguez, Fernando E. Casado, Roberto Iglesias, Carlos V. Regueiro, Adrián Nieto

**Affiliations:** 1 Situm Technologies S.L., Santiago de Compostela 15782, Spain; german.rodriguez@situm.es (G.R.); adrian.nieto@situm.es (A.N.); 2 CiTIUS, University of Santiago de Compostela, Santiago de Compostela 15782, Spain; fernando.estevez.casado@usc.es; 3 Department of Computer Engineering, University of A Coruña, A Coruña 15071, Spain; cvazquez@udc.es

**Keywords:** indoor-positioning, pedestrian dead reckoning, sensor fusion, step counting

## Abstract

Mobile phones are increasingly used for purposes that have nothing to do with phone calls or simple data transfers, and one such use is indoor inertial navigation. Nevertheless, the development of a standalone application able to detect the displacement of the user starting only from the data provided by the most common inertial sensors in the mobile phones (accelerometer, gyroscope and magnetometer), is a complex task. This complexity lies in the hardware disparity, noise on data, and mostly the many movements that the mobile phone can experience and which have nothing to do with the physical displacement of the owner. In our case, we describe a proposal, which, after using quaternions and a Kalman filter to project the sensors readings into an Earth Centered inertial reference system, combines a classic Peak-valley detector with an ensemble of SVMs (Support Vector Machines) and a standard deviation based classifier. Our proposal is able to identify and filter out those segments of signal that do not correspond to the behavior of “walking”, and thus achieve a robust detection of the physical displacement and counting of steps. We have performed an extensive experimental validation of our proposal using a dataset with 140 records obtained from 75 different people who were not connected to this research.

## 1. Introduction

We are immersed in a society of devices (mobile phones, tablets, wearables, etc.) that are transforming us at an incredible speed, changing the way we live or interact with each other. Not only do current generation devices incorporate increasingly better processors, but also high-end sensors previously unavailable. These progressive incorporation of sensors opens important opportunities towards the development of applications that use them in an increasing number of domains—healthcare, leisure, education, sport, social interaction, etc. One example of such is the use of the inertial sensors of the mobile phone to monitor human activity, in particular the action of walking. This information is very valuable for many applications, like biometric identification (e.g., recognition of the owner of the device by the way of walking) [1,2,3], medicine (detection of certain pathologies) [4], indoor localization, etc. In fact, in the particular case of pedestrian indoor localization, identifying the activity of walking using inertial sensors is essential, since alternatives like the Global Navigation Satellite System (GNSS) do not work indoors, and other sensor modalities such as WiFi or BlueTooth [5], infrared, ultrasound [6], vision based [7], magnetic field, audible sound or Ultra Wide Band (UWB), often provide a gross probabilistic estimation of the position, which can be significantly improved when combined with Pedestrian Dead Reckoning (PDR). PDR works with the estimated speed of a person to update the current position starting from a previous location. Different sensors and processing strategies have been applied to estimate this value of the speed: cameras for visual odometry, pressure sensors attached to the shoes using ultrasonic ranging between feet, inertial sensors, etc. [8]. Some of these approaches are undesirable since they require extra hardware attached to the body or, in the case of visual odometry, free line of sight of the person moving. On the contrary, inertial sensors (accelerometer, gyroscope and magnetometer) are more appealing, since they present fewer restrictions. Applying PDR when the sensors are placed on the body (foot, trunk, arm, etc. [9,10,11]) is more reliable and the processing of the sensor information is simpler than with the inertial sensors in the mobile phone. However, attaching sensors to the body or clothes very much restrains their applicability. Instead, working with the inertial sensors with which most mobile phones are already endowed is much more attractive, since most people carry these phones, and they can even run applications that use PDR-based techniques to offer localization services.

In this paper, we describe and analyze the performance of a novel strategy in the context of PDR, aimed at recognizing and counting steps given by any person carrying a mobile phone. The identification of human activities by using mobile phones and inertial sensors is much more complex than what it may seem. In the particular case of walking, it is relatively easy to recognize this activity and even count the steps when a person walks ideally with the mobile in the palm of his/her hand. Nevertheless, the situation gets much worse in real life, when the orientation of the mobile with respect to the body, as well as its location (hand, bag, pocket, ear, etc.), changes constantly while the person moves [12]. Mobile phones often experience a large variety of motions, which may have nothing to do with walking, but which can all produce similar patterns in the signal. This is what makes the recognition of this activity a complex task, especially when the performance is to be increased beyond a certain threshold.

Since we want no limitations about *where* or *how* the user carries the mobile, our proposal will estimate whether the person is walking before actually counting the steps. This will allow filtering chunks of useless signal and thus discarding false positives common in these kind of approaches. Obviously, with the proposal described in this paper, we tried to achieve a reliable and robust classification, able to achieve high performances regardless of how the device is being carried or its orientation. Nevertheless, we are aware that an idealistic 100% reliability is not feasible, which is why we also aim for a error-symmetric prediction (similar number of false positives and negatives). If this is true, these errors can cancel each other, so that the number of steps predicted by our proposal in a time interval will be very accurate. This will make our proposal very appropriate for indoor local navigation.

Even though there has been a significant amount of research in this subject, the current state-of-the-art solutions are far from optimal because they are either susceptible to false positives or require undesirable constraints or complexity. Additionally, the datasets used in most of the literature are not representative enough, i.e., they do not comprise the huge variability of situations in which an smartphone is often used/carried. In addition, in these datasets, the real steps walked by the persons are not individually labeled (one by one) to be used as ground truth. We have have actually built such a database (as we will describe in Section 4) and used it to perform an exhaustive analysis of the performance of each element of our proposal. Through the experimental results, and thanks to the use of our database, in which the steps are individually labeled, we will empirically demonstrate how our proposal fulfills all the objectives just described.

The rest of the paper is structured as follows: Section 2 describes the state of the art. Section 3 describes our proposal. Section 4 presents the database used for ground truth together with the experimental analysis of our proposal. The conclusions are presented in Section 5.

## 2. State of the Art

Thanks to the technological advances of the last years, it has been possible to integrate sensors of very small size in smartphones, like the inertial ones: accelerometer, gyroscope and magnetometer. Taking advantage of their presence, an emerging class of localization systems that use inertial sensors to perform dead reckoning on mobile phones is gaining attention. These systems have the advantage that very little physical infrastructure is required for them to run. Pedestrian Dead Reckoning works by estimating successive positions starting from a known location, based on a rough calculation of the direction and the distance walked. The direction can be obtained by the compass or the gyroscope. To determine the distance walked, a common solution is to count the number of steps and estimate their length. This is typically done analyzing the acceleration data [13,14,15], although other sensors have also been tried—Jimenez et al. [16] used the magnetometer in order to detect steps. In particular, they thresholded the magnitude of the signal that resulted after the removal of the DC component of the magnetic field—. In most studies, the position of the device is kept fixed (foot, trunk, arm, etc.) [9,10,11] because any changes in position may result in a loss of recognition performance. This is not desirable because people carry their smartphones in a variety of ways (hand, pocket, bag, etc.) and they do not keep it in a static position all the time. In recent years, especially with the popularization of smartphones, more research has been carried out on identification of steps and activity without restricting the position of the sensors.

A simple approach for step counting is the heuristic solution, present in many of the current commercial pedometer applications, and which consists of applying techniques such as the peak-valley detection to the magnitude of the acceleration [17,18]. This technique works by identifying peaks in the signal caused by the feet touching the floor (heel strikes) [14]. Thus, a step corresponds to a segment of signal in which there is a peak (local maximum which exceeds a threshold) followed by a valley (local minimum below a threshold). On the other hand, working with the magnitude of the acceleration avoids certain dependence on the orientation of the mobile phone. Another technique is zero crossing, which looks for the points at which the signal changes from positive to negative or the other way round [19]. However, these algorithms are susceptible to detect any motion as walking, hence they are prone to commit false positives. Due to this, another module responsible for filtering out is necessary, and, in real time, those parts of the signal that reflect some kind of movement in the mobile, but which have nothing to do with walking. This is a challenging task due to the high perceptual aliasing (i.e., the existence of many signals very similar but caused by different movements of the mobile or human actions).

A robust way of identifying the walking activity and counting the steps is by analyzing the shape of the inertial signals. In this case, it is assumed that it is possible to distinguish the activity of walking from that of not walking by simply observing the characteristic shape of the acceleration signal. This is mainly because, when walking, this signal shows a repetitive pattern, also known as step cycle. The event that is often used to mark the beginning of the cycle is the heel strike of the swing leg [2,3]. Then, it is necessary to find a match between the step cycle candidates and one or more patterns selected beforehand using a distance measure. Euclidean distance is a simple measure; however, it has been observed that it is very sensitive to distortion and noise and similar patterns can be separated by very large distances if the data items are not aligned [20]. A better alternative is to use warping distances, such as dynamic time warping (DTW) [21], longest common subsequence (LCSS) [22] or edit distance on real sequence (EDR) [20]. The main weakness of this approach is that, if the step candidates are misidentified during the signal division process, the subsequent matching with the reference patterns is compromised. In addition, there is a need to have a series of patterns that must be chosen beforehand and possibly manually.

Another way of dealing with this problem of step recognition is by extracting significant features from the inertial signals and using them to train a classifier. Susi et al. [18] identify the motion mode of the device (static, handheld texting, swinging, etc.) by extracting time and frequency domain features and applying a decision tree. (e.g., they classify a sequence as non-walking based on the periodicity and variance of the signal.). They use a different step detection algorithm for each motion mode, but all involve peak detection over the accelerometer or gyroscope signal. Finally, they apply a step length estimation using the step frequency and the height of the user. They report a good performance, but they only evaluated the performance of their step counting algorithm in restricted scenarios such as walking while texting and swinging. Bradjic et al. [23] conducted a survey in which they tested different step counting algorithms, and walking detection classifiers against a dataset of 130 sensor traces of 27 different users walking and performing different activities while carrying a smartphone. They concluded that neither of them performed better than a windowed peak detection, which presented a median error around 1.3%. However, it had a high rate of false positives when the device was moved without the user walking. They also obtained good classification rates using the standard deviation of the acceleration. Even though the accuracy reported is high, these algorithms will exhibit a high number of false positives when the phone is being moved, but the user is not walking.

There are also different commercial solutions for counting steps. As an example of this, we can mention Actigraph, which manufactures different activity monitoring devices frequently used in the medical field [24]. The main drawback is that additional hardware is required, and it is expensive. In addition, Eslinger et al. [25] obtained a solution that performs well when the user walks at a high speed, but the performance of which is significantly lower when the walking is slow. Another alternative used in medical literature is GAITRite1 [26]. This solution consists of a sensorized walkway that presents high precision in the detection of steps and activities. Nevertheless, this involves the deployment of hardware in the environment, and obviously a high cost and restricted areas of operation. Other alternatives, such as Fitbit, have proven to have a high number of false positives when performing activities different than walking [27].

We can conclude that even though there has been a lot of research in this subject, the current state of the art solutions are far from optimal because they are either susceptible to false positives or require undesirable constraints or complexity. Furthermore, most of the experimentation in the bibliography comprises only data of people walking, thus it is not clear how these algorithms would perform in terms of false positives when the person uses the phone without walking. Additionally, they tend to evaluate the performance of their proposals against the total traveled distance or the total detected steps, but there is no evaluation of whether a detected step is really a step or not (false positives and false negatives can cancel each other and mask the real performance of the system in short time intervals).

## 3. Walking Detection and Step Counting

**Figure 1:**
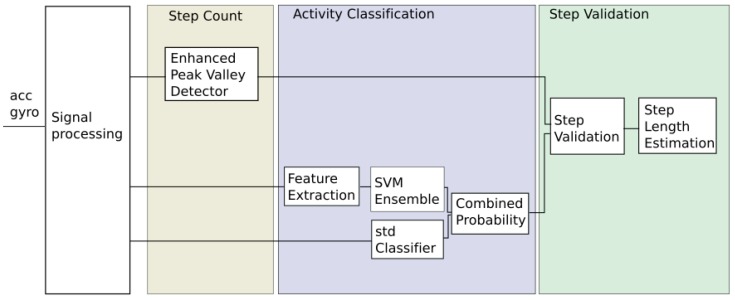
Our proposal to Pedestrian Dead Reckoning in a positioning system.

Figure 1 provides a schematic representation of our complete proposal. Basically it detects “step candidates”, i.e., a segment of signal that might reflect a step given by the person carrying the mobile. In parallel, supervised learning and Bayesian filtering are being used to recognize the walking activity. Hence, a step is validated and counted in those circumstances in which the detection of a step candidate coincides with the positive identification of the walking activity in the same signal segment. Next, we provide details of each one of the parts that make up our proposal.

### 3.1. Raw Signal Processing

The walking recognition and step counting is performed using the signals provided by the tri-axis accelerometer and the tri-axis gyroscope in a mobile phone, and which respectively measure the acceleration and the angular velocity of the device with respect to the navigation frame. The output of these sensors is a six-dimensional time series composed by the accelerometer output ${}^{S}{\vec{a}}_{t}\in R^{3}$ and the gyroscope output ${}^{S}{\vec{\omega }}_{t}\in R^{3}$, where *t* represents the temporal index of the signal. We choose a sampling frequency of 16 Hz for the accelerometer and 100 Hz for the gyroscope. The low sampling frequency in the case of the accelerometer responds to the need of achieving high performance with a low consumption: we want to run our algorithm on normal smartphones, and human walking frequency is usually between 1.5 Hz and 2.5 Hz, the reason why our sampling frequency of the accelerometer signal should be high enough.

At this stage, we carry out all the necessary transformations of the raw sensor data. Since the device can be carried at any position, first, we need to estimate its orientation with respect to an inertial frame. Then, we can estimate the vertical component of the acceleration that is being experienced by the mobile phone in this inertial reference system and, finally, we filter and center the resultant signal for noise reduction.

#### 3.1.1. Attitude Estimation

As we pointed out before, we need to know the attitude, or 3D orientation of the mobile phone with respect to an inertial frame, so that we can extract the vertical component of the acceleration experienced by the mobile. This signal will allow the identification of the heel strikes when walking. Some of the previous publications in walking recognition use the acceleration’s magnitude instead of the vertical component. Nevertheless, as the module is independent of a phone’s orientation, it is more failure prone in the presence of movements that have nothing to do with walking, thus increasing the rate of false positive detections.

To understand this stage, we must be aware of the existence of two reference systems: (1) a local reference system linked to the phone (also known as body frame or sensor frame). This local frame is defined relative to the device’s screen. (2) There is also an inertial reference system (Earth frame), the axes of which always point towards the same points (with respect to Earth). In the case of the inertial-Earth frame, we work with a frame analogous to the East North Up (ENU) coordinate system [28], in which the *x*-axis points toward the East, the *y*-axis points towards the North Magnetic Pole and the *z*-axis is pointing in the opposite direction of the gravitational force. The accelerometer and gyroscope readings are provided in the body frame, and therefore it is convenient to project them into the inertial-Earth frame in order to estimate the movement of the person who carries the mobile. Hence, it is necessary to know the orientation (attitude) of the mobile with respect to the inertial-Earth frame.

In order to represent this orientation, we use quaternions [29,30] because of their many advantages over other representations. A quaternion is a four-dimensional vector that represents the relative orientation between two coordinate frames *B* and *A*, as a rotation of an angle θ around a three-dimensional axis *r*:

(1) $${}_{B}^{A}q=\left[q_{0}\;q_{1}\;q_{2}\;q_{3}\right]=\left[cos\frac{\theta }{2},\;r_{x}sin\frac{\theta }{2},\;r_{y}sin\frac{\theta }{2},\;r_{z}sin\frac{\theta }{2}\right],$$

where ${}_{B}^{A}q$ is the normalized quaternion that represents the orientation of a frame *B* relative to a frame *A* [30]. Following this notation, we will use ${}_{E}^{S}q_{t}$ to refer to the current value of the quaternion that represents the orientation of frame *E* (Earth frame), relative to the frame *S* (local/Sensor frame). This quaternion represents the current state of the mobile phone. We will use an Extended Kalman Filter (EKF) [31] to estimate these quaternions. Our EKF will work with a process mode. that is represented as the evolution of the state due to the rotation of the mobile detected with gyroscope. Since the gyroscope measures the rate of angular velocity, it can be used to determine the orientation:

(2) $${}_{E}^{S}{\hat{q}}_{t}^{-}={}_{E}^{S}{\hat{q}}_{t-1}^{+}+\frac{1}{2}\left({}_{E}^{S}{\hat{q}}_{t-1}^{+}⊗{}^{S}{\vec{\omega }}_{t}\right)\;\Delta t,$$

where ⊗ is the quaternion product, ${}^{S}{\vec{\omega }}_{t}=\left(0,{}^{S}\omega_{x,t},{}^{S}\omega_{y,t},{}^{S}\omega_{z,t}\right)$ is the vector that arranges the readings (angular velocities) provided by the tri-axial gyroscope at the current instant, *t*. ${}_{E}^{S}{\hat{q}}^{-}$ is the a priori estimate of the state before the current sensor observations are processed, whereas ${}_{E}^{S}{\hat{q}}^{+}$ is the a posteriori estimate. Therefore, given an initial orientation, the information provided by the gyroscope can be integrated to determine the device’s change in position.

The gyroscope has a high error rate (its data drifts over time), is unstable, and low angular velocities might not be properly registered. Because of all this, and to compensate for all these errors, the EKF uses a *measurement model* to compute the posterior estimate ${}_{E}^{S}{\hat{q}}^{+}$, Equation (2). Let us consider ${}^{S}{\vec{a}}_{t}$ as the vector that arranges the current tri-axial accelerometer readings: ${}^{S}{\vec{a}}_{t}=\left\{0,{}^{S}a_{x,t},{}^{S}a_{y,t},{}^{S}a_{z,t}\right\}$, the observation model of the EKF works on the basis that when the magnitude of the current value of the accelerometer signal ($∥{}^{S}{\vec{a}}_{t}∥$) is close to Earth’s gravity (*g*)—which would mean that the mobile phone is not being affected by other forces—the accelerometer should measure only Earth-gravity in the local device frame. In this case, the projection of the unit gravity vector in the Earth frame, ${\vec{u}}_{G}=\vec{G}/∥G∥=\left\{0,0,0,1\right\}$, into the local reference system (body frame), should coincide with the information detected by the tri-axial accelerometer ${}^{S}{\vec{a}}_{t}$. This projection of ${\vec{u}}_{G}$ into the local reference system can be computed as:

(3) $${}_{E}^{S}{\hat{q}}_{t}^{-*}⊗{\vec{u}}_{G}{⊗}_{E}^{S}{\hat{q}}_{t}^{-},$$

where ${}_{E}^{S}{\hat{q}}_{t}^{-*}$ is the conjugate of ${}_{E}^{S}{\hat{q}}_{t}^{-}$ [30]. According to this, when ${\vec{u}}_{G}$ is rotated using the current estimation of the quaternion ${}_{E}^{S}{\hat{q}}_{t}^{-}$ (Equations (2) and (3)), we should obtain the same values as those currently provided by the accelerometer ${}^{S}{\vec{a}}_{t}$. In fact, this difference amongst the predicted values of the accelerometer and the ones observed is precisely what the EKF uses to correct the a priori estimate of the quaternion:

(4) $${}_{E}^{S}{\hat{q}}_{t}^{+}={}_{E}^{S}{\hat{q}}_{t}^{-}+K_{t}\left[{}_{E}^{S}{\hat{q}}_{t}^{-*}⊗{\vec{u}}_{G}⊗{}_{E}^{S}{\hat{q}}_{t}^{-}-{}^{S}{\vec{a}}_{t}\right],$$

where *K* is the Kalman gain, a dynamic parameter that weights the importance between the predicted state ${}_{E}^{S}{\hat{q}}_{t}^{-}$ and the information carried out by the observations ${}^{S}{\vec{a}}_{t}$, and which depends on the process noise and the observation noise [32]. The EFK together with the quaternions has been already used in other works for the estimation of orientation [19,33].

#### 3.1.2. Estimation of the Vertical Linear Acceleration

Since we know the attitude of the phone, we can now obtain the vertical component of the linear acceleration experienced by the mobile in the Earth reference system. To do so, we just remove the gravity force:

(5) $$lacc_{t}{=}_{E}^{S}q_{t}⊗{}^{S}{\vec{a}}_{t}{⊗}_{E}^{S}q_{t}^{*}-\vec{G},$$

where ${}^{S}{\vec{a}}_{t}$ is the vector that arranges the accelerometer readings. The first term of the previous equation (Equation (5)) represents the projection of the information gathered by the accelerometer into the Earth frame. $\vec{G}=\left(0,0,0,9.81\right)$ is the vector representation of the gravity force in the Earth frame. The vertical linear acceleration, $lacc_{z,t}$ (i.e., the vertical acceleration experienced by the mobile once the gravity has been removed), is the last component of the vector represented in Equation (5), $lacc_{t}=\left(lacc_{0,t},\;lacc_{1,t},\;lacc_{2,t},\;lacc_{3,t}=lacc_{z,t}\right)$).

#### 3.1.3. Signal Characteristics

**Figure 2:**
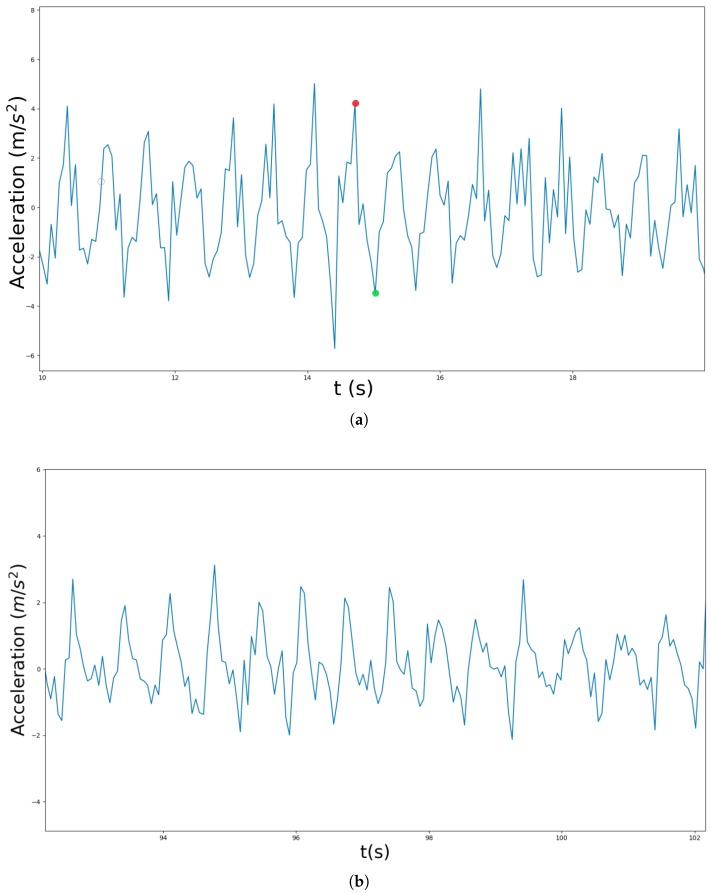
Vertical linear acceleration sampled at 16 Hz. (**a**) signal obtained when the user walking while holding its phone; (**b**) signal obtained when the mobile phone is being moved by the user but without walking.

At this point, we have isolated the vertical component of acceleration and eliminated the effect of gravity from it. When a person walks carrying a phone in its hand, and in the absence of other stimuli, the signal received (i.e., the vertical component of the acceleration in the Earth reference system) is often similar to a sinusoidal pattern shown in Figure 2a. Consequently, the signal shown in this figure can be considered as the characteristic signal generated by a person walking at a relatively low frequency [34]. In this signal, each step taken by the person is identified by the segment formed by a local peak followed by a valley. Hence, in this scenario, it might seem that a Peak Valley algorithm should be enough to identify the steps walked by a person. However, things usually are far from this ideal case, since there is a problem of perceptual aliasing, i.e., very similar signals to the one shown in Figure 2a can be obtained when the mobile phone is being used in a natural way, but when the person is not walking at all (Figure 2b). This makes the identification and segmentation of the signal into walking versus non-walking segments a difficult problem. Section 3.3 describes the way we have addressed this issue.

In order to achieve a satisfactory identification of steps, the vertical linear acceleration should be centered in zero as seen in Figure 2, but some devices may have a bias in the readings of the accelerometer that might prevent this centering. Non centered signals can lead to errors in the step detection algorithms. To ensure that this does not happen in our case, we apply a high pass filter to center the signal and thus remove any possible DC offset:

(6) $$y_{t}=\alpha_{h}y_{t-1}+\alpha_{h}\left(x_{t}-x_{t-1}\right),$$

where $x_{t}$ is the input (vertical linear acceleration signal), $y_{t}$ the filtered signal, $\alpha_{h}=\frac{RC}{RC+\Delta t}$ and $RC=\frac{1}{2\pi f_{c}}$ and $f_{c}$ the cutoff frequency (0.5 Hz).

Finally, we apply a low pass filter to remove the noise from the signal:

(7) $$y_{t}=\alpha_{l}x_{t}+\left(1-\alpha_{l}\right)\left(y_{t-1}\right),$$

where *x* is the input (i.e., the already centered acceleration after applying Equation (6)), *y* the filtered signal, $\alpha_{l}=\frac{\Delta t}{RC+\Delta t}$ and $RC=\frac{1}{2\pi f_{c}}$.

### 3.2. Step Detection

Step or gait detection can be achieved in different ways. One typical approach is to use a peak detector [35] to identify events, like heel strikes, where the impacts of the feet are reflected in the vertical acceleration signal. Other approaches involve exploiting the cyclic and repetitive nature of walking, and hence using properties such as the signal auto-correlation [36]. In our case, we have used an enhanced Peak Valley detector that locates local extremes in the signal. In this case, a step corresponds to a segment of signal in which there is a peak (local maximum which exceeds a threshold) followed by a valley (local minimum below a threshold). On the other hand, the time elapsed from the previous detected step to the new step-candidate must be above a valid walking period for it to be accepted. This is due to the fact that humans commonly walk within a low range of frequencies [34].

However, this peak valley algorithm is susceptible to detect any motion produced within the expected range of frequencies, and hence is prone to commit false positives and, on the other hand, usually has problems detecting changes in the walking speed [23]. Due to this, we consider the use of another module responsible for filtering out necessary, and, in real time, those parts of the signal which reflect some kind of movement in the mobile, but which have nothing to do with walking. This is a challenging task due to the high perceptual aliasing (i.e., the existence of many signals very similar but caused by different movements of the mobile or human actions).

### 3.3. Walking Recognition

In this section, we describe a module aimed at detecting when the user is walking, and thus discard noisy signals and filter out the false positives detected by the peak-valley algorithm described in the previous section. This module is strictly necessary to reach a symmetric error: on one side, the number of false positives will decrease since a step will be counted if, and only if, it is detected by the peak-valley algorithm and the activity of walking is recognized in the same signal segment by this module. On the other side, the inclusion of this module will have the collateral effect of increasing the number of false negatives, as a consequence of the misclassification of signals (although, as we will see, the rate increase of false negatives will be low). As we will see in the experimental results, the number of false positives and false negatives will tend to be very similar, thus canceling each other and making this proposal very suitable for pedestrian dead reckoning and indoor positioning.

We use supervised learning and Bayesian filtering [37] to differ walking from non-walking sequences in the signal. To do so we define the state $x_{t}$ as a vector which reflects whether the user is walking or not. Bayesian filtering involves the recursive application of a *prediction* and an *update* stages. The predictive distribution of the state $x_{t}$ can be computed by the equation:

(8) $$p\left(x_{t}|y_{1:t-1}\right)=p\left(x_{t}|x_{t-1}\right)p\left(x_{t-1}|y_{1:t-1}\right),$$

where $y_{1:t-1}$ is the history of measurements up to the time t-1. Therefore, as we can see, we work with a very simple dynamic model in which the only important element is 2×2 transition matrix $p(x_{t}|x_{t-1})$, the elements of which have been learned by an inductive process using a training data set. This transition matrix basically introduces a certain amount of hysteresis in the process: i.e., if the user was detected as walking in the previous time interval, there is a not null probability that the user is still walking at the current instant, despite the fact that the activity might not be identified in the current signal segment. Only after a certain time-lapse without recognizing the walking activity does this module set the probability of walking as null. Something similar happens in the contrary case.

Regarding the *update* stage, the Bayes’ rule will be applied to estimate the posterior distribution of the state $x_{t}$, starting from the current measurement $y_{t}$:

(9) $$p\left(x_{t}|y_{1:t}\right)=\alpha p\left(y_{t}|x_{t}\right)p\left(x_{t}|y_{1:t-1}\right),$$

where α is a normalizing constant. We write $y_{t}$ to refer to the current observation. In our case, we work with the overlapping sliding window, i.e., segments of signal and not instantaneous sensor readings. This means that $y_{t}$ represents the observed signal ($lacc_{z}$) in a window, while $y_{t}$ and $y_{t-1}$ represent two different windows that overlap. To compute the probability $p(y_{t}|x_{t}),$ we will merge an ensemble of SVMs together with a logistic estimator that works over the standard deviation of the signal. Regarding the SVM, we opted for an ensemble instead of a single SVM. We work with an ensemble because we have an unbalanced training dataset, with more walking sequences than non-walking sequences; therefore, we decided to use Bagging, oversampling randomly and with replacement from the minority class [38]. The outputs of all the SVMs that are part of the ensemble were merged to get a probability value:

(10) $$p_{svm}\left(y_{t}|x_{t}\right)\propto \frac{1}{n}\sum_{i=1}^{n}z_{svm}^{i},$$

where *n* is the number of classifiers in the ensemble, and $z_{svm}$ represents the output of the *i*th member of the committee. The inputs of these SVMs are feature vectors computed over windows of 2.5 s that overlap 0.5 s. Using feature vectors instead of raw data can reduce the number of input elements and improve the generalization ability. We performed a study to identify which features were the most relevant for detecting walking sequences from accelerometer and gyroscope data. First, we collected all the features relevant to activity classification from the literature both in temporal and frequency domains [39,40,41]. Then, we analyzed the relevance of each feature with Recursive Feature Elimination (RFE) [42]. The selected features in the time domain are: (1) The Signal Magnitude Area (SMA), (2) Maximum value for each axis of the accelerometer and gyroscope, (3) Standard deviation for each axis of accelerometer and gyroscope, and (4) Correlation between axes *y* and *z* of the accelerometer.

The signal magnitude area (SMA) is computed as:

(11) $$SMA=\frac{1}{w}\left(\sum_{i=1}^{w}|acc_{x}^{i}|+\sum_{i=1}^{w}|acc_{y}^{i}|+\sum_{i=1}^{w}\left|acc_{z}^{i}\right|\right),$$

where $acc_{x}$, $acc_{y}$ and $acc_{z}$ are the acceleration signals perceived from each axis of the accelerometer, and *w* is the size of the window. Finally, the formula for the correlation is:

(12) $$corr\left(acc_{y},acc_{z}\right)=\frac{cov(acc_{y},acc_{z})}{\sigma_{acc_{y}}\sigma_{acc_{z}}},$$

where $cov(acc_{y},acc_{z})$ is the covariance and $\sigma_{acc_{y}}$ and $\sigma_{acc_{z}}$ the standard deviation in each axis. Regarding the features in the frequency domain, after applying the Fast Fourier Transform (FFT), we work with (1) the FFT frequency bins up to 4 Hz for each axis of the acceleration signal; (2) the Dominant frequency in each axis which can be directly estimated from the FFT bin with the maximum value; and (3) Power Spectral Density Entropy (PSD) [43]:

(13) $$PSD=-\sum_{i=1}^{N}p_{i}\ln p_{i},$$

where $p_{i}$ is the power spectral density function:

(14) $$p_{i}=\frac{\frac{1}{N}{\left|X\left(w_{i}\right)\right|}^{2}}{\sum_{i}\frac{1}{N}{\left|X\left(w_{i}\right)\right|}^{2}}$$

and where $X(w_{i})$ is the FFT of a signal, $w_{i}$ is a frequency bin, and *N* is the number of frequency bins. This feature can be interpreted as a measurement of the uncertainty in the frequency domain.

Despite obtaining good performance with this ensemble of SVMs, due to the difficulty of the task, we decided to use a logistic estimator to reduce the number of misclassification. The results shown in [23] support the idea of applying a threshold over the standard deviation of the acceleration to identify walking sequences in most scenarios. Therefore, we constructed an estimator of the probability $p(y_{t}|x_{t})$ as logistic function of the standard deviation of the vertical linear acceleration $lacc_{z}$:

(15) $$p_{std}\left(y_{t}|x_{t}\right)\propto \left\{\begin{array}{cc} \frac{1}{1+e^{-k(\sigma_{lacc_{z}}-\beta_{0})}}, & \mathrm{if}\;\sigma_{lacc_{z}}<th,\\ \frac{1}{1+e^{k(\sigma_{lacc_{z}}-\beta_{1})}}, & \mathrm{otherwise}, \end{array}\right.$$

where $\sigma_{lacc_{z}}$ is the standard deviation of the vertical linear acceleration (computed over sliding windows of one second), *k* and $\beta_{0}$ and $\beta_{1}$ are constants set experimentally that define the steepness and midpoints of the curves respectively, and th is a threshold set in the midpoint between $\beta_{0}$ and $\beta_{1}$. The idea behind this function is that typical walking sequences should have a standard deviation within a certain range of values. The logistic functions ensure smooth transitions.

Finally, we combined the results obtained with both the ensemble of SVMs, and the logistic function of the standard deviation:

(16) $$p\left(y_{t}|x_{t}\right)=p_{svm}\left(y_{t}|x_{t}\right)p_{std}\left(y_{t}|x_{t}\right).$$

### 3.4. Estimation of the Distance Traveled by the User

**Figure 3:**
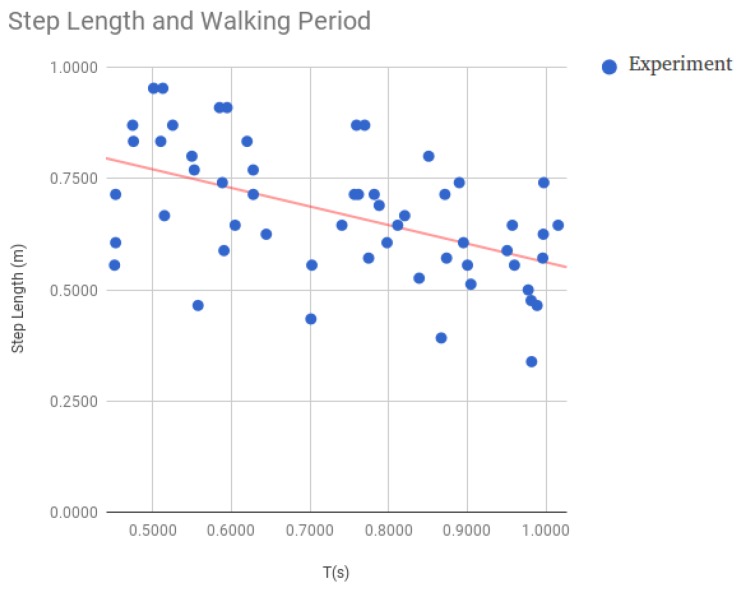
Relationship between walking period and step length.

At this point, we already have information about the steps the user is taking; however, we still need to estimate the length of the steps in order to compute the displacement of the user. Due to the fact that there is a relationship between the step length and walking frequency [8], we decided to model the length of the step considering only the walking period [35]. Estimating this period from consecutive valid steps is straightforward; nevertheless, for the matter of robustness, we work with the average of the last *N* detected steps. To get the relationship amongst the walking period and the step length, we asked several people to walk along a 20 m hallway at a constant speed. We instructed them to repeat this walk at different paces. For each record, we calculated the average step length (from the total distance and the number of steps walked). We also estimated the average walking period. Figure 3 plots both variables for the data obtained experimentally showing that there is a clear relationship between them. Finally, we applied Linear Least Squares to relate the step length with the walking period.

Thus, when the peak-valley (Section 3.2) detector detects a step, we estimate the distance traveled by simply applying:

(17) $$d_{t}=l_{t}\cdot P\left(walk_{t}\right),$$

where $l_{t}$ is the length estimated for the step, and $P(walk_{t})$ is the probability that the user is walking. This value estimated from the estate $x_{t}$ described in Section 3.3 in Equation (9). Therefore, the system will count only steps when the peak-valley algorithm detects an step and the walking recognition confirms that the user is moving.

## 4. Experimental Analysis and Results

We want to evaluate the performance of our proposal in realistic situations. To this end, we carried out the following experiments: First, we evaluated the step identification and counting when different people—mostly unrelated with our research to avoid bias—moved in a completely free manner, hence in a natural way, regarding both the walking manner and also the way they carried the mobile phone (Section 4.2). Then, we evaluated the performance of our proposal to estimate the distance traveled when a person is walking (Section 4.3). Finally we tested the robustness of our proposal to different hardware (different mobile phones, Section 4.4).

In order to carry out these experiments, and analyze the performance of the proposal described in this paper, we require a way of obtaining the ground truth of the steps given by the user, i.e., the real number of steps walked by the person carrying the mobile. In some of the experiments, a visual counting might be enough, but, in some other experiments, where many users take part, a data set is collected, and where not only do we want to know the number of steps but also their timestamps—time values corresponding to each step—the visual counting is insufficient, prone to errors. In these cases, we had to make use of an automatic strategy (explained in Section 4.1.)

### 4.1. Obtaining the Ground Truth

As we pointed out in the previous section, we are going to collect data to analyze the number of steps given, due to which we need a ground truth. Most articles in the bibliography evaluate the performance of their algorithms only taking into account the total number of steps detected per experiment. Nevertheless, in our case, we opted for labeling every single step, in order to analyze the performance of each part of our proposal, as well as the canceling of false positives and false negatives thanks to the symmetry in the error. Hence, in our ground truth, each step is perfectly identified together with its timestamp, i.e., the moment in which the step is given. One option to obtain this ground truth could be manual counting. However, this method is unfeasible and prone to errors if we want to perform many experiments involving different people moving freely. Moreover, manual counting would not allow us to get the timestamps of the steps in an easy way. There are commercial step-counting solutions that perform well when the user walks, even though some of them are still susceptible to detect false positives [27], or they involve sensorized environments that constrain the freedom of movement of the user [44]. Nevertheless, we want to emphasize that, even though there might be commercial solutions available that would be valid to get the ground truth, we still opted for building our own mechanism due to two reasons: (1) our solution will require attaching inertial sensors to the legs (like some of the commercial solutions); as we will see, this will allow the detection of the movement of legs of the users unambiguously, and hence will provide a very reliable ground truth. On the other hand, (2) thanks to the use of our own sensors and software, we can get precise information about each single step walked by the user, and synchronize the readings provided by the sensors attached to the legs, with the signal that is being detected in the mobile carried by the person in the experiment and which is running the proposal described in this paper. This synchronization will allow a deep analysis of our proposal.

**Figure 4:**
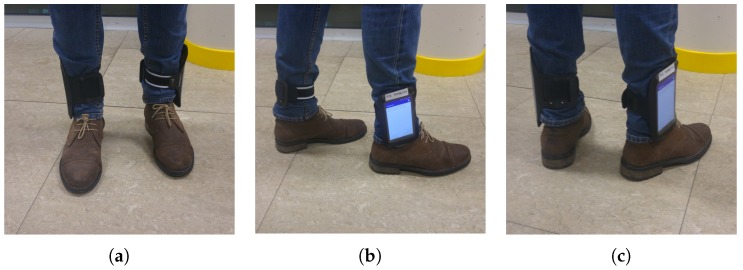
Sports armbands holding the mobiles of the legs. (**a**) Frontal view; (**b**) side view; (**c**) rear view.

Attaching inertial sensors (accelerometers) to the legs, unlike the personal smartphone that can be carried on the hand, the pocket, the backpack, etc., removes uncertainty regarding when the person is walking. When these sensors detect the presence of important forces, we know that are due to the direct movement of the legs. Therefore, these sensors give precise information of when the user walks by the fact of being located on the legs. We have developed a system that uses two extra smartphone-based IMUs in the legs instead of any other low-cost integrated IMU. We attach smartphones on the legs instead of other low-cost IMU sensors, as the mobile phone can be considered a computer that can facilitate some tasks, such as real-time signal processing or synchronization. Hence, we tied these extra devices with sports armbands to the legs of the people who participated in our experiments, as shown in Figure 4.

**Figure 5:**
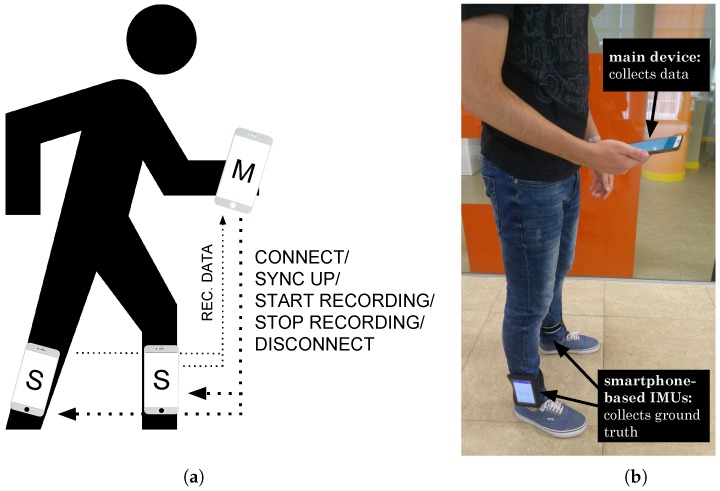
Communication and synchronization between devices. (**a**) representation of the master-slave architecture; (**b**) volunteer obtaining data and its corresponding ground truth.

We developed an Android application that was installed on all three smartphones. The devices worked in a synchronized manner storing information from the accelerometer, gyroscope and magnetometer sensors. The devices interact with each other following a master/slave communication protocol. Thus, the main mobile (the master) controls the other two devices in the legs (the slaves), as shown in Figure 5a. For all the devices to be synchronized as accurately as possible, all of them make a query to the same time server (time.google.com) using the library TrueTime [45] in order to get an atomic time, which allows later to compare information of different devices without problems of alteration of precedence.

**Figure 6:**
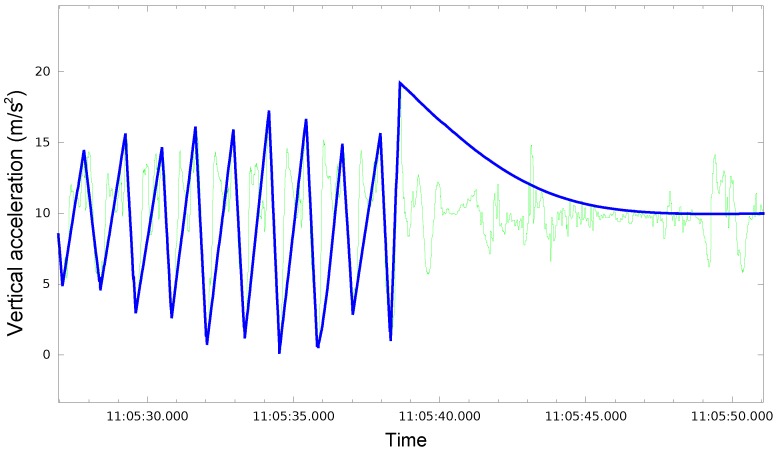
Graphic representation of the ground truth (thicker and darker line) over the signal of the vertical component of acceleration in the phone (thinner and clearer line).

Figure 6 shows a graphic representation of the ground truth over the signal of the vertical component of acceleration in the main mobile phone. Each peak-valley sequence in the ground truth signal is equivalent to one step, so it is easy to identify when the user is really walking and when the main device is experiencing accelerations due to actions different from walking.

**Figure 7:**
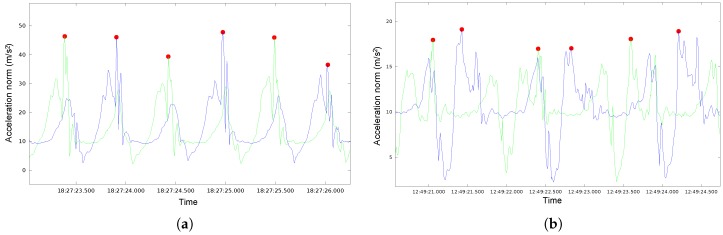
Peak detection (thick points) combining the signals of the two feet (blue and green lines) in an ideal situation (**a**) and a non ideal situation (**b**).

The ground truth was calculated using only the data from the devices in the legs. First, we got two signals, one for each device fixed in each leg, using the acceleration module at each moment. In order to detect possible steps, we used a peak-valley algorithm [14], and then we matched the signals obtained with both mobiles on the legs. In this way, we can ideally detect each step in the signals coming from both legs, as shown in Figure 7a. Nevertheless, depending on various conditions, such as the person performing the record, speed and way of walking, there are diverse situations that require special processing and attention: the signals detected with both legs might be shifted (Figure 7b), making it difficult to determine when it is the same or a different step. It is also possible to miss a peak in one of the legs due to an occasional weak signal. Finally, it can also happen that the peak of greatest intensity is recorded in the foot opposite to the one that has given the step. Nevertheless, we want to emphasize that our system has been designed to cope robustly with all these situations.

Given the large number of records performed in which the users walked under very different and greatly varied characteristics, we assume that there might be some steps missing in the ground truth. In order to somehow analyze to what extent this happens, we have also validated the ground truth through manual counting and visual inspection, counting steps of a significant part of the dataset (28%). This analysis allows us to limit the error committed in our ground truth in ±2%, that is, between one and two steps per record.

### 4.2. Step Detection and Walking Recognition Performance Analysis

In this first experiment, we wanted to evaluate the performance of our proposal when different people use it; to this aim, we have built a large dataset composed of a total of 140 records carried out by 75 different people. The vast majority of them (70, specifically) were volunteers with no links or connection with the research described in this paper. We have proceeded in this way in order to ensure that the data were not biased. Thus, in each record, the participant walked under natural conditions, freely or following some basic premises, while the inertial information of its movements was recorded and processed. Each volunteer walked, on average, about 2 min, giving around 110 steps. The dataset contains a variety of records that can be classified according to various criteria, such as the volunteer who performed the record, speed and way of movement: walking straight, climbing stairs, walking freely, etc. Another possible classification considers the position of the mobile, or the way in which it was carried: in the hand, in the pocket, in the backpack, etc. In all of the experiments in the dataset, the smartphone used was a BQ Aquaris E5 (BQ, Madrid, Spain). We labeled in this dataset each step taken with its timestamp using the method described in Section 4.1.

We have applied a matching algorithm to compare *step by step* the output of our proposal and the ground truth. We have analyzed the performance of the Peak Valley algorithm, also the walking recognition working as a classifier, and finally the complete system. Therefore, we have detailed information about true step detections (true positives), false positives, as well as false negatives. We can not compute the true negatives for the peak valley algorithm, since we speak about steps that do not appear in the ground truth and which are not detected by the Peak Valley algorithm. In the case of the walking recognition working as a classifier, the true negatives only reflect those false steps detected by the Peak Valley algorithm but discarded by the walking recognition working as a classifier. We want to emphasize that this is one of the first articles in the literature (as far as we know) where there is such an exhaustive comparison, *step by step*.

We evaluated the performance of our algorithm dividing the dataset as follows: (1) Global performance, using the whole dataset. (2) Performance when the user walks freely with the phone in its hand. (3) Performance when the user walks freely with the phone in its pocket. (4) Performance in hybrid sequences, in which the user walks freely using the phone and changing its position (e.g., walks with it in the hand and then puts it back in the pocket). (5) Performance when the user barely walks but is using and moving the phone.

Table 1 shows the confusion matrices for each kind of experiment, while Table 2 shows the total amount of steps detected by the peak valley and by the complete system compared to the ground truth. To better understand these tables, we explain below the results obtained for the complete dataset (Table 1a and the first column of Table 2). The number of steps in the ground truth is 13,810. From these steps, the Peak Valley algorithm detects correctly 13,104, misses 706 real steps and counts 1640 incorrect steps. Therefore, the total amount of steps detected by the peak valley is 14,744, which are the candidate steps used as input for the classifier (the sum of the values in the confusion matrix of the classifier is equal to 14,744). From these candidate steps, the classifier detects 13,803 steps as correct, from which 12,544 are true positives and 1259 false positives. It also discards 941 steps, 381 of them being true negatives and 560 false negatives. The right side of the table shows the confusion matrix of the complete system. In this matrix, the steps detected as true (true positives + false positives) are the same as that in the classifier matrix. The false negatives are the sum of the steps missed by the peak valley (706) plus the the steps missed by the classifier (560). Finally, the number of true negatives is unknown, since it would be the sum of the 381 true negatives of the classifier plus the true negatives of the Peak Valley.

Peak Valley performs well when the user is really walking but otherwise counts a high amount of false positives, as was expected. Our system discards part of the false positives, but it also misses some of the real steps, thus achieving an error symmetric approach. Our estimation of the steps missed by the ground truth leads us to conclude that both errors tend to be roughly the same. Since the error committed by the classifier is reasonable, the false positives and false negatives cancel each other out, thus achieving a high accuracy (as it is shown in Table 2). In this table, we can see how the total amount of steps counted by our complete system is significantly closer to the ground truth than the number counted with the Peak Valley algorithm only. Therefore, we think that this makes our proposal suitable for tasks such as Pedestrian Dead Reckoning and, in combination with other techniques, for indoor location.

Our system filters out noise in the signal, which is particularly relevant in sequences in which the user uses the phone without barely walking. However, it still has a significant amount of false positives, a problem that we will address in future research.

### 4.3. Analysis of the Estimated Distance

Besides the analysis of the performance of our proposal when recognizing steps, we also carried out experiments to evaluate the accuracy of the estimations made by our system regarding the distance walked by the users.

**Figure 8:**
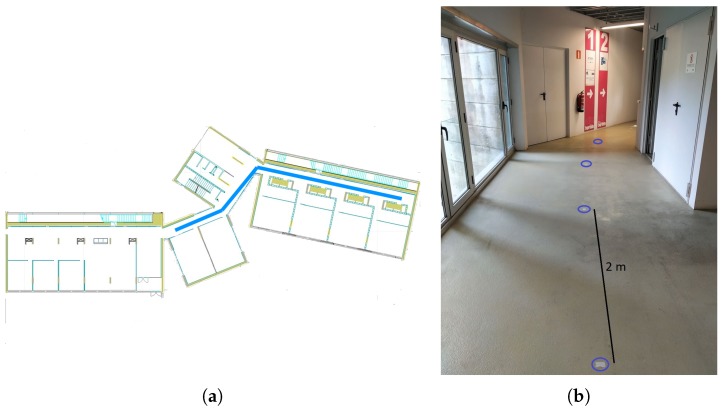
(**a**) path followed by the people taking part in the experiment aimed for the analysis of the performance of our proposal at estimating the distance travelled by a person walking; (**b**) marks on the ground every 2 m placed to indicate the path that must be followed during the experiment.

We run 20 experiments with 10 users in which they had to walk along a predefined path of 44 m. This path is shown in Figure 8a. During the experiment, there were marks on the ground every two meters to indicate to the users the path that they had to follow Figure 8b. The users walked following the route and carrying a phone that recorded the data. We run our system using these data and compared the distance estimated with our proposal versus the ground truth. It should be noted that the ground truth may have an error of approximately ±1 m, due to some possible experimental artifacts: the last step taken by the user does not have to necessarily coincide with the last mark on the floor, and there can be a little deviation between the path followed by a person and the one marked on the ground, etc. The device used in these experiments was a BQ Aquaris E5.

**Figure 9:**
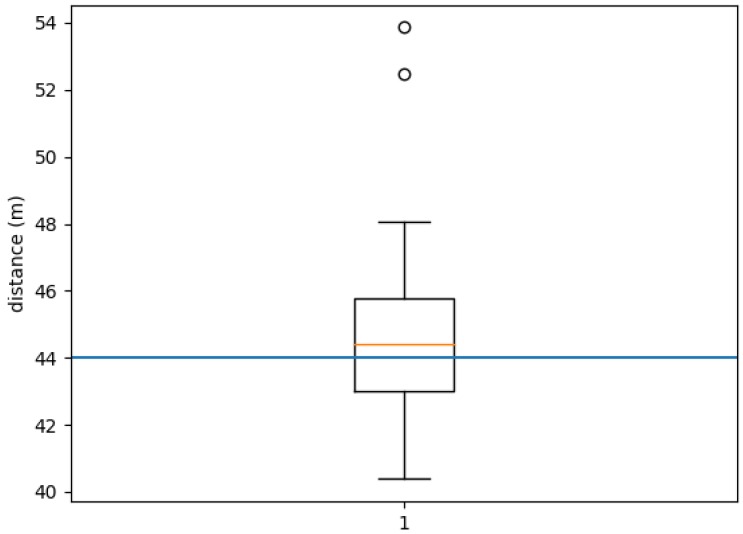
Boxplot with the distances estimated by our proposal for the 44 m long path.

Figure 9 and Table 3 show the results of the experiment. In Figure 9, we can see a boxplot with the distances estimated by our proposal. The blue line represents the ground truth. It can be seen that the median is slightly above the ground truth of 44 m and the percentiles Q1 and Q3 only deviate from it around 2 m. There are two outliers that identified a distance greater than 10 m above the real one. It is interesting to note that both belong to the same person.

As it was previously shown in Figure 3, there is a relationship between step length and the walking period, which is taken into a account by our model. Nevertheless, our proposal does not consider the influence of other variables like the user height, etc. The only way we foresee including those other variables is by means of personalized models that can integrate particular information of the user carrying the mobile. Nevertheless, considering the outcome of this experiment, our proposal performs well enough and it does not seem necessary to include this other information to improve the step size estimation.

### 4.4. Analysis of the Independence of Our Proposal to the Hardware Being Used

In this experiment, we will study the impact of the hardware—model of the mobile phone being used—in the performance of our proposal. Hardware differences generally involve different acquisition frequencies, differences in the amount of noise in the signal, and some devices can even have a bias in one or more of the sensor axes. These signal differences should not affect our algorithm thanks to the subsampling and filtering of the signal, the use of robust features, an ensemble of classifiers or Bayesian filtering, etc., with which our proposal should generalize well. However, we performed experiments with different smartphones, in order to analyze their impact on the outcome of our proposal.

We took different models of smartphones at the same time and walked while running our system. If our hypothesis is correct, the number of steps detected by each smartphone should be very similar for each experiment.

We performed four experiments using five terminals at the same time. In the first two, we carried all of the telephones in the same hand, and, in the next two, in the pocket. In all of them, we took 60 steps. The results can be seen in Table 4. For the experiments in the hand, there are hardly any variations. In those experiments in which the mobiles are placed in the pockets, experiments (3 and 4), there is a slightly higher variation regarding the number of steps counted with our proposal. Nevertheless, even in this case, the difference is not important, since the maximum difference between the steps counted for the different mobiles is three steps. Looking at the results, we can say that there are certain differences, especially when the mobile phone is in the pocket, but they should not represent a serious threat.

## 5. Conclusions

In this paper, we describe a robust solution to detect the displacement of a person carrying a mobile phone. Our proposal processes the information provided by the inertial sensors of the phone to estimate the number of steps walked by the person as well as their length. Due to the necessity of allowing the user to move freely with no bounds about the position or way of carrying the phone, we had to include the recognition of the walking activity to reduce the number of mistakes that a classic peak valley algorithm would commit. The recognition of the walking activity filters out those parts of the signal that are due to movements of the phone that have nothing to do with the act of walking. This is a complex task due to the existence of a high aliasing, i.e., we get very similar signals for many different movements of the mobile, which makes the filtering out extremely difficult. In this work, we collected an extensive dataset with a large amount of different people walking naturally under different circumstances in order to validate our proposal. We have carried out an extensive analysis of the performance comparing step by step the ground truth with the output of our system. Although further improvements can be carried out in an attempt to increase the performance, we believe that the combined use of our proposal with other positioning systems (WiFi, for example) would allow the achievement of ever more robust estimations of the movement of the person, although this will be part of our future research. 

## Figures and Tables

**Table 1:** Confusion matrices of the Peak Valley detector, the walking recognition working as a classifier over the candidate steps detected by the Peak Valley, and the Complete System, for each subset of data. Columns show the output of the system while the rows show the output of the ground truth (GT).

	(a) Complete Dataset										
		**Peak Valley**				**Classifier**			**Complete System**		
		**True**	**False**			**True**	**False**			**True**	**False**
GT	true	13,104 (85%)	706 (5%)		true	12,544 (85%)	560 (4%)		true	12,544 (83%)	1266 (8%)
	false	1640 (10%)	-		false	1259 (8%)	381 (3%)		false	1259 (8%)	-
	**(b) Hand**										
		**Peak Valley**				**Classifier**			**Complete System**		
		**True**	**False**			**True**	**False**			**True**	**False**
GT	true	5775 (92%)	213 (4%)		true	5630 (92%)	145 (2%)		true	5630 (90%)	358 (6%)
	false	311 (5%)	-		false	259 (4%)	52 (1%)		false	259 (4%)	-
	**(c) Pocket**										
		**Peak Valley**				**Classifier**			**Complete System**		
		**True**	**False**			**True**	**False**			**True**	**False**
GT	true	2848 (83%)	195 (6%)		true	2693 (83%)	155 (5%)		true	2693 (80%)	350 (10%)
	false	398 (12%)	-		false	320 (10%)	78 (2%)		false	320 (10%)	-
	**(d) Hybrid**										
		**Peak Valley**				**Classifier**			**Complete System**		
		**True**	**False**			**True**	**False**			**True**	**False**
GT	true	2547 (84%)	163 (5%)		true	2410 (84%)	137 (5%)		true	2410 (81%)	300 (10%)
	false	321 (11%)	-		false	255 (9%)	66 (2%)		false	255 (9%)	-
	**(e) Not Walking**										
		**Peak Valley**				**Classifier**			**Complete System**		
		**True**	**False**			**True**	**False**			**True**	**False**
GT	true	137 (26%)	22 (4%)		true	114 (23%)	23 (4%)		true	114 (30%)	45 (12%)
	false	368 (70%)	-		false	226 (45%)	142 (28%)		false	226 (59%)	-

**Table 2:** Total steps in the ground truth, detected by the Peak Valley (PV) detector and detected by the whole system (PV with the walking recognition working as a classifier).

Total Steps					
	**Dataset**	**Hand**	**Pocket**	**Hybrid**	**Not Walking**
GT	13,810	5988	3043	2710	159
PV	14,744	6086	3246	2868	505
PV + C	13,803	5889	3013	2665	340

**Table 3:** Statistical values corresponding to the distances estimated with our proposal.

	Distance (m)
Average	45.11
Standard Deviation	3.31
Maximum	53.8
Minimum	40.38

**Table 4:** Steps detected in four experiments running our proposal with different smartphones.

	Hand		Pocket	
	**Experiment 1**	**Experiment 2**	**Experiment 3**	**Experiment 4**
BQ Aquaris E5	58	57	58	60
Samsung Galaxy A2	58	58	59	59
Xiaomi Mi A2	58	58	59	62
Motorola Moto G6 plus	58	58	61	60
OnePlus 2	58	58	60	59

## References

[1] Lu H., Huang J., Saha T., Nachman L. Unobtrusive gait verification for mobile phones. Proceedings of the 2014 ACM International Symposium on Wearable Computers.

[2] Ren Y., Chen Y., Chuah M.C., Yang J. (2015). User verification leveraging gait recognition for smartphone enabled mobile healthcare systems. IEEE Trans. Mob. Comput..

[3] Teixeira T., Jung D., Dublon G., Savvides A. PEM-ID: Identifying people by gait-matching using cameras and wearable accelerometers. Proceedings of the Third ACM/IEEE International Conference on Distributed Smart Cameras.

[4] Dutta S., Chatterjee A., Munshi S. (2009). An automated hierarchical gait pattern identification tool employing cross-correlation-based feature extraction and recurrent neural network based classification. Expert Syst..

[5] Liu H., Darabi H., Banerjee P., Liu J. (2007). Survey of wireless indoor positioning techniques and systems. IEEE Trans. Syst. Man Cybern. Part C (Appl. Rev.).

[6] Randell C., Muller H. (2001). Low cost indoor positioning system. International Conference on Ubiquitous Computing.

[7] Mautz R., Tilch S. Survey of optical indoor positioning systems. Proceedings of the 2011 International Conference on Indoor Positioning and Indoor Navigation (IPIN).

[8] Harle R. (2013). A survey of indoor inertial positioning systems for pedestrians. IEEE Commun. Surv. Tutor..

[9] Kourogi M., Ishikawa T., Kurata T. A method of pedestrian dead reckoning using action recognition. Proceedings of the 2010 IEEE/ION Position Location and Navigation Symposium (PLANS).

[10] Vathsangam H., Emken A., Spruijt-Metz D., Sukhatme G.S. Toward free-living walking speed estimation using Gaussian Process-based Regression with on-body accelerometers and gyroscopes. Proceedings of the 2010 4th International Conference on Pervasive Computing Technologies for Healthcare.

[11] Steinhoff U., Schiele B. Dead reckoning from the pocket-an experimental study. Proceedings of the 2010 IEEE International Conference on Pervasive Computing and Communications (PerCom).

[12] Yang J., Lu H., Liu Z., Boda P.P. (2010). Physical activity recognition with mobile phones: Challenges, methods, and applications. Multimedia Interaction and Intelligent User Interfaces.

[13] Stirling R., Collin J., Fyfe K., Lachapelle G. An innovative shoe-mounted pedestrian navigation system. Proceedings of the European Navigation Conference GNSS.

[14] Lee H.H., Choi S., Lee M.J. (2015). Step detection robust against the dynamics of smartphones. Sensors.

[15] Bayat A., Pomplun M., Tran D.A. (2014). A study on human activity recognition using accelerometer data from smartphones. Procedia Comput. Sci..

[16] Jimenez A.R., Seco F., Prieto C., Guevara J. A comparison of pedestrian dead-reckoning algorithms using a low-cost MEMS IMU. Proceedings of the 2009 IEEE International Symposium on Intelligent Signal Processing.

[17] Qian J., Ma J., Ying R., Liu P., Pei L. An improved indoor localization method using smartphone inertial sensors. Proceedings of the 2013 International Conference on Indoor Positioning and Indoor Navigation (IPIN).

[18] Susi M., Renaudin V., Lachapelle G. (2013). Motion mode recognition and step detection algorithms for mobile phone users. Sensors.

[19] Goyal P., Ribeiro V.J., Saran H., Kumar A. Strap-down pedestrian dead-reckoning system. Proceedings of the 2011 International Conference on Indoor Positioning and Indoor Navigation (IPIN).

[20] Chen L., Özsu M.T., Oria V. Robust and fast similarity search for moving object trajectories. Proceedings of the 2005 ACM SIGMOD International Conference on Management of Data.

[21] Berndt D.J., Clifford J. Using dynamic time warping to find patterns in time series. Proceedings of the KDD Workshop.

[22] Vlachos M., Kollios G., Gunopulos D. Discovering similar multidimensional trajectories. Proceedings of the 18th International Conference on Data Engineering.

[23] Brajdic A., Harle R. Walk detection and step counting on unconstrained smartphones. Proceedings of the 2013 ACM International Joint Conference on Pervasive and Ubiquitous Computing.

[24] Sasaki J.E., John D., Freedson P.S. (2011). Validation and comparison of ActiGraph activity monitors. J. Sci. Med. Sport.

[25] Esliger D.W., Probert A., Connor S.G., Bryan S., Laviolette M., Tremblay M.S. (2007). Validity of the Actical accelerometer step-count function. Med. Sci. Sports Exerc..

[26] Webster K.E., Wittwer J.E., Feller J.A. (2005). Validity of the GAITRite^®^ walkway system for the measurement of averaged and individual step parameters of gait. Gait Posture.

[27] O’Connell S., Olaighin G., Quinlan L.R. (2017). When a step is not a step! Specificity analysis of five physical activity monitors. PLoS ONE.

[28] Grewal M.S., Weill L.R., Andrews A.P. (2007). Global Positioning Systems, Inertial Navigation, and Integration.

[29] Shuster M.D. (1993). A survey of attitude representations. Navigation.

[30] Madgwick S.O. (2010). An Efficient Orientation Filter for Inertial and Inertial/Magnetic Sensor Arrays.

[31] Simon D. (2006). Optimal State Estimation: Kalman, H Infinity, and Nonlinear Approaches.

[32] Thrun S. (2002). Probabilistic robotics. Commun. ACM.

[33] Sabatini A.M. (2011). Kalman-Filter-Based Orientation Determination Using Inertial/Magnetic Sensors: Observability Analysis and Performance Evaluation. Sensors.

[34] Zijlstra W., Hof A.L. (1997). Displacement of the pelvis during human walking: Experimental data and model predictions. Gait Posture.

[35] Li F., Zhao C., Ding G., Gong J., Liu C., Zhao F. A reliable and accurate indoor localization method using phone inertial sensors. Proceedings of the 2012 ACM Conference on Ubiquitous Computing— UbiComp ’12.

[36] Rai A., Chintalapudi K.K., Padmanabhan V.N., Sen R. Zee: Zero-effort crowdsourcing for indoor localization. Proceedings of the 18th Annual International Conference on Mobile Computing and Networking.

[37] Durrant-Whyte H.F., Henderson T.C., Siciliano B., Khatib O. (2008). Multisensor data fusion. Springer Handbook of Robotics.

[38] Breiman L. (1996). Bagging predictors. Mach. Learn..

[39] Preece S.J., Goulermas J.Y., Kenney L.P.J., Howard D., Meijer K., Crompton R. (2009). Activity identification using body-mounted sensors—A review of classification techniques. Physiol. Meas..

[40] Yang J.Y., Wang J.S., Chen Y.P. (2008). Using acceleration measurements for activity recognition: An effective learning algorithm for constructing neural classifiers. Pattern Recognit. Lett..

[41] Bernecker T., Graf F., Kriegel H., Moennig C., Dill D., Tuermer C. (2012). Activity Recognition on 3D Accelerometer Data.

[42] Guyon I., Weston J., Barnhill S., Vapnik V. (2002). Gene selection for cancer classification using support vector machines. Mach. Learn..

[43] Zhang A., Yang B., Huang L. Feature extraction of EEG signals using power spectral entropy. Proceedings of the 2018 International Conference on BioMedical Engineering and Informatics.

[44] Thomas M., Jankovic J., Suteerawattananon M., Wankadia S., Caroline K.S., Vuong K.D., Protas E. (2004). Clinical gait and balance scale (GABS): Validation and utilization. J. Neurol. Sci..

[45] Gopal K. (2016). TrueTime for Android. https://github.com/instacart/truetime-android.

